# An Atypical Case of Late-Onset Wolfram Syndrome 1 without Diabetes Insipidus

**DOI:** 10.3390/ijerph19042473

**Published:** 2022-02-21

**Authors:** Luciana Rigoli, Valerio Caruso, Concetta Aloi, Alessandro Salina, Mohamad Maghnie, Giuseppe d’Annunzio, Olga Lamacchia, Giuseppina Salzano, Fortunato Lombardo, Giuseppe Picca

**Affiliations:** 1 Department of Human Pathology of Adulthood and Childhood G. Barresi, University of Messina, 98125 Messina, Italy; giuseppina.salzano@unime.it (G.S.); fortunato.lombardo@unime.it (F.L.); 2 Psychiatry 2 Unit, Clinical and Experimental Medicine Department, University of Pisa, 56126 Pisa, Italy; valeriocaruso79@gmail.com; 3 Pediatric Clinic, LABSIEM (Laboratory for the Study of Inborn Errors of Metabolism), IRCCS Institute Giannina Gaslini, 16147 Genoa, Italy; aloigre@alice.it (C.A.); alessandrosalina@yahoo.it (A.S.); mohamadmaghnie@gaslini.org (M.M.); giuseppedannunzio@ospedale-gaslini.ge.it (G.d.); 4 Unit of Endocrinology and Diabetology, Department of Medical and Surgical Sciences, University of Foggia, 71100 Foggia, Italy; olga.lamacchia@unifg.it (O.L.); piccadrp@hotmail.com (G.P.)

**Keywords:** wolfram syndrome 1, atypical phenotype, DIDMOAD, *WFS1* mutations

## Abstract

Wolfram syndrome 1, a rare autosomal recessive neurodegenerative disease, is caused by mutations in the *WFS1* gene. It is characterized by diabetes insipidus, diabetes mellitus, optic atrophy, and deafness (DIDMOAD), and other clinical manifestations such as urological and neurological disorders. Here we described the case of a patient with an atypical late-onset Wolfram syndrome 1 without DI. Our WS1 patient was a c.1620_1622delGTG (p.Trp540del)/c.124 C > T (p.Arg42*) heterozygous compound. The p.Arg42* nonsense mutation was also found in heterozygosity in his sister and niece, both suffering from psychiatric disorders. The p.Arg42* nonsense mutation has never been found in WS1 and its pathogenicity is unclear so far. Our study underlined the need to study a greater number of WS1 cases in order to better understand the clinical significance of many *WFS1* variants.

## 1. Introduction

Wolfram syndrome 1 (WS1; OMIM 222300) is a rare, progressive, neurodegenerative disease with autosomal recessive inheritance [1]. The prevalence of WS1 in the general population ranges from 1/777,000 [2] to 1/54,478 [3] in diverse ethnic groups. The carrier frequency is 1/354 [4]. Non-insulin dependent, non-autoimmune diabetes mellitus (DM), and optic atrophy (OA) are the main criteria for the diagnosis of WS1 [5]. DM usually occurs during the first decade of life, and OA before 15 years of age. DM and OA are often associated with diabetes insipidus (DI) and deafness (D), which are usually diagnosed in the second decade of life of WS1 patients. Therefore, WS1 is also called DIDMOAD [2,6]. Other common clinical features of WS1 include urinary tract anomalies, and neuropsychiatric and endocrine disorders [7]. The acronym DIDMOADUD has been suggested, as urinary tract abnormalities are very frequent in patients with WS1 [8]. WS1 is characterized by early mortality, with an average age of death around 30 (range 25–49 years). The main cause of death is central respiratory failure, due to brain stem atrophy [2,9].

Many studies have shown that WS1 is caused by mutations in the *WFS1* gene, which is located on chromosome 4p16.1. *WFS1* includes eight exons and encodes wolframin, an 890 amino acid protein with nine putative transmembrane domains, mainly located in the endoplasmic reticulum (ER) membrane [10]. Wolframin plays a key role in both the pathway of ER stress and in regulating calcium levels in cells. It is highly expressed in the pancreas, brain, heart, and muscles, while it is poorly expressed in the liver and kidneys [11,12]. *WFS1* pathogenic variations have been found in 75–90% of WS1 patients [13], and it has been shown that they cause high levels of ER stress, and severe alterations of β pancreatic and neurological cells up to cell death [14]. There is a broad spectrum of *WFS1* mutations causing WS1, such as missense, frameshift, nonsense, and spice mutations, predominantly localized in exon 8 [15]. Patients with a clinical picture of WS1 are homozygotes or compound heterozygotes for *WFS1* mutations as they are carriers of mutations in both alleles of the gene. On the other hand, heterozygous subjects, that is, monoallelic carriers of *WFS1* mutations, may have an increased risk of psychiatric diseases and suicide, as well as of DM and sensorineural D [15].

Herein, we described the case of a patient with WS1 who showed an atypical clinical picture.

## 2. Methods

### 2.1. Case Report

**Figure 1:**
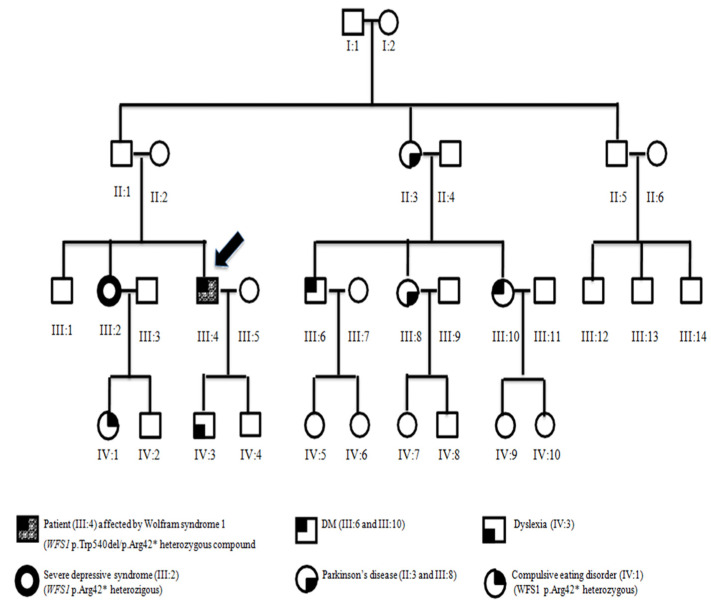
Familial pedigree of patient affected by Wolfram syndrome 1.

A 48-year-old male presented to the Endocrinology and Metabolic Diseases Unit of OORR University Hospital of Foggia (Italy) with a clinical history of DM, D, and OA. He had non-insulin dependent, non-autoimmune DM, which was diagnosed at the age of three and, thus, he underwent insulin therapy. Bilateral sensorineural D was diagnosed at age 31 and bilateral OA at age 35. With the patient, we performed physical examination, laboratory tests, complete neurological and eye exams, audiology and vestibular tests, urologic evaluation, and neuroimaging. All laboratory and instrumental tests were performed with the WS1 patient to exclude pathologies that could have masked DI to confirm the true absence of DI. Figure 1 shows the proband’s familial pedigree. Blood samples were obtained from the patient, his sister (III:2), and his niece (IV:1).

### 2.2. Molecular Analysis

Genetic testing was performed to confirm the suspicion of WS1. DNA from the proband, his sister, and his niece was extracted from whole blood using the High Pure PCR Template Preparation Kit (Roche, Mannheim, Germany). Unfortunately, blood samples from other relatives were not available. The exons and flanking regions of *WFS1* (OMIM:626201; NM_006005.3) were amplified by polymerase chain reaction (PCR) using previously described primers [16]. Automatic sequencing of the amplified samples was performed by an automated fluorescent sequencing method (Big Dye Terminator Kit v1.1, Applied Biosystems). The products were separated on an ABI PRISM sequencing apparatus 3730 (Applied Biosystems). All variations were validated by sequencing both DNA strands of three independent PCR products. The sequence variants were considered mutations when they (a) caused a non-conservative amino acid change, (b) were absent in 300 ethnically matched control chromosomes, and (c) affected phylogenetically conserved residues. Other DNA variations that did not fulfill these criteria were considered polymorphisms.

### 2.3. Bioinformatics

All the identified *WFS1* variants were checked for novelty utilizing HGMD (http://www.hgmd.cf.ac.uk/ac/index.php) (accessed on 15 May 2019), Exac (http://exac.broadinstitute.org/) (accessed on 15 May 2019), EVS (http://evs.gs.washington.edu/EVS/) (accessed on 15 May 2019), *dbSNP* (http://www.ncbi.nlm.nih.gov./projects/SNP/) (accessed on 15 May 2019), and VarSome (https://varsome.com/) (accessed on 15 May 2019). To better define the potential pathogenicity of the *WFS1* variants, computational analyses were performed with Mutation Taster and Sift, two free tools that assess whether a DNA sequence variant alters the function of a specific protein and, therefore, whether it has a disease-causing potential.

## 3. Results and Discussion

### 3.1. Clinical Data

The parents of our WS1 patient were not consanguineous (II:1 and II:2). They had three children, two boys and one girl, with one of them (III:4) showing WS1 clinical features. Family history has shown that some members were suffering from pathologies such as Parkinson’s disease (subjects II:3 and III:8), adult-onset DM (III:6 and III:10), obsessive-compulsive personality traits with eating disorders (IV:1), and dyslexia (IV:3) (Figure 1).

### 3.2. WFS1 Mutation Analysis

The WS1 patient, his sister, and his niece were subjected to genetic analysis. Automated sequencing of *WFS1* was performed, including all exons and exon–intron boundaries. Genetic testing allowed us to detect heterozygous compound mutations [c.1620_1622delGTG (p.Trp540del)] + [c.124 C > T (p.Arg42*)] in our WS1 patient; his sister and niece were heterozygous for c.124 C > T (p.Arg42*).

We were the first to describe the *WFS1* c.1620_1622delGTG (p.Trp540del) deletion [16]. It was found in a female with WS1, in which DM and OA appeared early at the age of 6 and 8 years, respectively. The deletion was in exon 8, where most of the variants causative of WS1 have been found. It was an in-frame deletion causing the loss of one tryptophan residue (p.Trp540del) and was in the coding region for the sixth domain of the wolframin transmembrane region [16]. In the case described above, the WS1 patient was a p.Cys505Tyr/p.Trp540del heterozygous compound in which the expression of a deletion and of a nonsense mutation resulted in a severe phenotype [16]. A severe early onset type of WS1 caused by c.1620_1622delGTG (p.Trp540del) deletion in homozygosis has also been described in a North African family in which there were two siblings affected by WS1. A 22-year-old male manifested the complete clinical picture of DIDMOAD, myoclonic epilepsy, and pontocerebellar ataxia. His 11-year-old sister had only insulin-dependent, non-autoimmune DM, probably because the complete WS1 phenotype had not yet fully manifested at her young age. Both siblings were homozygous for c.1620_1622delGTG (p.Trp540del) deletion [17]. Therefore, it appeared that in the two cases described here, p.Trp540del causes a severe clinical picture of WS1. Therefore, these studies showed that the *WFS1* p.Trp540del leads to serious alterations in the functional expression of wolframin and, thus, to severe WS1 phenotypes. In our WS1 patient, we also found the nonsense variant c.124 C > T (p.Arg42*) in exon 4 of *WFS1*, within a nucleotide sequence encoding the N-terminal region of wolframin [18]. This *WFS1* mutation leads to a premature termination codon at position 42, resulting in a truncated or absent protein.

The question is how much did p.Arg42* contribute to the manifestation of the clinical picture of WS1? It is difficult to understand as the c.124 C > T (p.Arg42*) mutation has been differently classified between databases for human gene mutations. The VarSome database describes *WFS1* p.Arg42* as polymorphism, and it is reported in *dbSNP* as *rs71530923*. ClinVar and ACMG classify it as “likely pathogenic or pathogenic” and “pathogenic”, respectively. The nonsense change has been found in 0.02% (1/8540) of European American chromosomes by the NHLBI exome sequencing project (http://evs.gs.washington.edu/EVS/) (accessed on 25 January 2022). However, we believe that the *WFS1* p.Arg42* mutation may be considered as pathogenic, not only because of its low frequency in the general population, but also because it was associated with some clinical disorders (https://www.ncbi.nlm.nih.gov/clinvar/) (accessed on 15 May 2019). On the other hand, it was observed that heterozygotes carriers of *WFS1* variants could develop sensorineural hearing loss and/or DM [19,20,21]. It is not surprising that the *WFS1* p.Arg42* mutation is pathogenic as it leads to a near total absence of wolframin. Furthermore, homozygous WS1 patients have never been identified for this mutation. Could the *WFS1* p.Arg42* mutation be lethal in utero? Other studies are needed to clarify the role of this *WFS1* mutation.

In our study, we described for the first time a patient suffering from an unusual clinical picture of WS1 in which the p.Arg42* mutation was in the heterozygosity compound with the p.Trp540del variant of *WFS1* (p.Trp540del/p.Arg42* genotype). The p.Arg42* variant has not previously been identified in WS1 patients, and, in our case, the p.Trp540del/p.Arg42* genotype was associated to an uncommon phenotype of WS1. Moreover, our patient started to suffer from DM at age 3, while D and OA occurred many years later, at age 31 and age 35, respectively. Unusually, the patient’s symptom triad (DM, D, and OA) was not accompanied by DI. Moreover, why did it take so long from the diagnosis of DM until the other symptoms of WS1 appeared? It cannot be assumed that D and OA have not been diagnosed earlier as they are complications which are too serious to go unnoticed. We hypothesized that in our patient who was a p.Trp540del/p.Arg42* heterozygous compound, the residual functional expression of wolframin resulted in a less severe WS1 phenotype. This would explain the absence of DI, or of urological and neuropsychiatric symptoms in the patient’s clinical picture, and a later onset of D and OA. We have also found that the patient’s sister and niece were both heterozygous for the p.Arg42* mutation, one suffering from severe depressive syndrome (III:2), and the other from compulsive eating disorders. The patient’s son (IV:3) was suffering from dyslexia, but unfortunately, his DNA was not available. Heterozygotes for *WFS1* mutations may manifest some disorders such as psychiatric illness, DM, or sensorineural deafness, but not the complete clinical picture of WS1, which, on the other hand, is only found in the presence of biallelic mutations of the *WFS1* [15].

Other atypical cases of WS1 have been described, and in some of them it was shown that the same *WFS1* mutation could cause different clinical pictures. Recently, Wilf-Yarkoni et al. described a milder, late-onset WS1 phenotype in patients with the *WFS1* p.Arg558Cys mutation [22]. However, previously, Lieber et al. had identified this mutation in a case of WS1 in which the patient had diabetes mellitus, diffuse brain atrophy, autonomic neuropathy, optic nerve atrophy, and severe amnestic syndrome. Further genetic analysis performed on this patient had identified multiple heteroplasmic deletions of mitochondrial DNA [23]. This highlights that phenotypic heterogeneity occurs in patients with the same mutation, probably due to genetic and environmental backgrounds.

The clinical and genetic characteristics of our WS1 patient and his relatives allowed some assumptions to be made: the nonsense mutation *WFS1* p.Arg42* had a pathogenic role, causing, together with p.Trp540del, the clinical picture of WS1 with late onset. Moreover, subjects heterozygous for this variant could be predisposed to neuropsychiatric diseases. This is in addition to the already known predisposition to deafness and diabetes mellitus.

## 4. Conclusions

We reported the case of a patient with *WFS1* p.Trp540del/p.Arg42* genotype and late onset of clinical manifestations of WS1. *WFS1* p.Arg42* mutation was reported in *dbSNP* as *rs71530923* or classified as “likely pathogenic or pathogenic”. In our study, we have shown for the first time that the *WFS1* p.Arg42* nonsense mutation in heterozygosity compound with the p.Trp540del caused WS1. Moreover, two relatives of the WS1 patient who were heterozygous for the p.Arg42* variant had psychiatric disorders. Our new observation is in addition to the already known predisposition to deafness and diabetes mellitus, which has been associated with the *WFS1* p.Arg42* variant [19,20,21]. Genotype–phenotype correlation in WS1 is very difficult as the number of WS1 patients is very low. Therefore, further studies are needed in order to clarify the clinical significance of the many *WFS1* mutations.

## Data Availability

Not applicable.

## References

[1] Wolfram D.J., Wagener H.P. (1938). Diabetes mellitus and simple optic atrophy among siblings: Report of four cases. Mayo Clin. Proc..

[2] Barrett T.G., Bundey S.E., Macleod A.F. (1995). Neurodegeneration and diabetes: UK nationwide study of Wolfram (DIDMOAD) syndrome. Lancet.

[3] Lombardo F., Salzano G., Di Bella C., Aversa T., Pugliatti F., Cara S., Valenzise M., De Luca F., Rigoli L. (2014). Phenotypical and genotypical expression of Wolfram syndrome in 12 patients from a Sicilian district where this syndrome might not be so infrequent as generally expected. J. Endocrinol. Investig..

[4] Nakamura A., Shimizu C., Nagai S., Taniguchi S., Umetsu M., Atsumi T., Wada N., Yoshioka N., Ono Y., Tanizawa Y. (2006). A novel mutation of WFS1 gene in a Japanese man of Wolfram syndrome with positive diabetes-related antibodies. Diabetes Res. Clin. Pract..

[5] Chaussenot A., Bannwarth S., Rouzier C., Vialettes B., Mkadem S.A., Chabrol B., Cano A., Labauge P., Paquis-Flucklinger V. (2011). Neurologic features and genotype-phenotype correlation in Wolfram syndrome. Ann. Neurol..

[6] Marshall B.A., Permutt M.A., Paciorkowski A.R., Hoekel J., Karzon R., Wasson J., Viehover A., White N.H., Shimony J.S., Manwaring L. (2013). Washington University Wolfram Study Group. Phenotypic characteristics of early Wolfram syndrome. Orphanet J. Rare Dis..

[7] Rigoli L., Bramanti P., Di Bella C., De Luca F. (2018). Genetic and clinical aspects of Wolfram syndrome 1, a severe neurodegenerative disease. Pediatr. Res..

[8] Urano F. (2016). Wolfram Syndrome: Diagnosis, Management, and Treatment. Curr. Diabetes Rep..

[9] Rigoli L., Lombardo F., Di Bella C. (2011). Wolfram syndrome and WFS1 gene. Clin. Genet..

[10] Inoue H., Tanizawa Y., Wasson J., Behn P., Kalidas K., Bernal-Mizrachi E., Mueckler M., Marshall H., Donis-Keller H., Crock P. (1998). A gene encoding a transmembrane protein is mutated in patients with diabetes mellitus and optic atrophy (Wolfram syndrome). Nat. Genet..

[11] Gómez-Zaera M., Strom T.M., Rodríguez B., Estivill X., Meitinger T., Nunes V. (2001). Presence of a major WFS1 mutation in Spanish Wolfram syndrome pedigrees. Mol. Genet. Metab..

[12] Takeda K., Inoue H., Tanizawa Y., Matsuzaki Y., Oba J., Watanabe Y., Shinoda K., Oka Y. (2001). WFS1 (Wolfram syndrome 1) gene product: Predominant subcellular localization to endoplasmic reticulum in cultured cells and neuronal expression in rat brain. Hum. Mol. Genet..

[13] Rigoli L., Di Bella C. (2012). Wolfram syndrome 1 and Wolfram syndrome 2. Curr. Opin. Pediatr..

[14] Fonseca S.G., Fukuma M., Lipson K.L., Nguyen L.X., Allen J.R., Oka Y., Urano F. (2005). WFS1 is a novel component of the unfolded protein response and maintains homeostasis of the endoplasmic reticulum in pancreatic beta-cells. J. Biol. Chem..

[15] De Heredia M.L., Clèries R., Nunes V. (2013). Genotypic classification of patients with Wolfram syndrome: Insights into the natural history of the disease and correlation with phenotype. Genet. Med..

[16] Colosimo A., Guida V., Rigoli L., Di Bella C., De Luca A., Briuglia S., Stuppia L., Salpietro D.C., Dallapiccola B. (2003). Molecular detection of novel WFS1 mutations in patients with Wolfram syndrome by a DHPLC-based assay. Hum. Mutat..

[17] Giuliano F., Bannwarth S., Monnot S., Cano A., Chabrol B., Vialettes B., Delobel B., Paquis-Flucklinger V. (2005). Wolfram syndrome in French population: Characterization of novel mutations and polymorphisms in the WFS1 gene. Hum. Mutat..

[18] Gharanei S., Zatyka M., Astuti D., Fenton J., Sik A., Nagy Z., Barrett T.G. (2013). Vacuolar-type H^+^-ATPase V1A subunit is a molecular partner of Wolfram syndrome 1 (WFS1) protein, which regulates its expression and stability. Hum. Mol. Genet..

[19] Franks P.W., Rolandsson O., Debenham S.L., Fawcett K.A., Payne F., Dina C., Froguel P., Mohlke K.L., Willer C., Olsson T. (2008). Replication of the association between variants in WFS1 and risk of type 2 diabetes in European populations. Diabetologia.

[20] Sandhu M.S., Weedon M.N., Fawcett K.A., Wasson J., Debenham S.L., Daly A., Lango H., Frayling T.M., Neumann R.J., Sherva R. (2007). Common variants in WFS1 confer risk of type 2 diabetes. Nat. Genet..

[21] Ohata T., Koizumi A., Kayo T., Shoji Y., Watanabe A., Monoh K., Higashi K., Ito S., Ogawa O., Wada Y. (1998). Evidence of an increased risk of hearing loss in heterozygous carriers in a Wolfram syndrome family. Hum. Genet..

[22] Wilf-Yarkoni A., Shor O., Fellner A., Hellmann M.A., Pras E., Yonath H., Shkedi-Rafid S., Basel-Salmon L., Bazak L., Eliahou R. (2021). Mild Phenotype of Wolfram Syndrome Associated with a Common Pathogenic Variant Is Predicted by a Structural Model of Wolframin. Neurol. Genet..

[23] Lieber D.S., Calvo S.E., Shanahan K., Slate N.G., Liu S., Hershman S.G., Gold N.B., Chapman B.A., Thorburn D.R., Berry G.T. (2012). Targeted exome sequencing of suspected mitochondrial disorders. BMC Med. Genet..

